# Evaluation of two commercial kits and two laboratory-developed qPCR assays compared to LAMP for molecular diagnosis of malaria

**DOI:** 10.1186/s12936-022-04219-1

**Published:** 2022-06-27

**Authors:** Azza Bouzayene, Rizwana Zaffaroullah, Justine Bailly, Liliane Ciceron, Véronique Sarrasin, Sandrine Cojean, Nicolas Argy, Sandrine Houzé, Valentin Joste, the French National Malaria Reference Centre study group, Adela Angoulvant, Anne Pauline Bellanger, Antoine Huguenin, Anthony Marteau, Agnes Durand, Céline Tournus, Céline Nourrisson, Céline Malassigne, Cécile Garnaud, Caroline Lohmann, Edith Mazars, Emilie Sitterle, Eric Dannaoui, Françoise Botterel, Guillaume Desoubeaux, Ghania Belkadi, Isabelle Salimbeni, Jean Philippe Lemoine, Luce Landraud, Louise Basmaciyan, Loic Favennec, Marie Fleur Durieux, Marie Laure Darde, Milene Sasso, Marc Thellier, Naima Dahane, Nathalie Fauchet, Nathalie Bourgeois, Odile Eloy, Odile Fenneteau, Pascale Penn, Pauline Caraux Paz, Roseanne Lavergne, René Nabias, Sorya Belaz, Sylvain Mermond, Samia Hamane, Sébastien Larréché, Sylvain Clauser, Stéphane Lastere, Yaye Senghor, Yohann Le Govic

**Affiliations:** 1 National Malaria Reference Centre, AP-HP, Hôpital Bichat - Claude Bernard, 46 Rue Henri Huchard, 75018 Paris, France; 2 University of Paris Cité, IRD, MERIT, 75006 Paris, France

**Keywords:** Malaria, *Plasmodium*, *P. falciparum*, Molecular diagnosis, qPCR, LAMP, Bio-Evolution, Biosynex

## Abstract

**Background:**

Malaria is an infectious disease considered as one of the biggest causes of mortality in endemic areas. This life-threatening disease needs to be quickly diagnosed and treated. The standard diagnostic tools recommended by the World Health Organization are thick blood smears microscopy and immuno-chromatographic rapid diagnostic tests. However, these methods lack sensitivity especially in cases of low parasitaemia and non-falciparum infections. Therefore, the need for more accurate and reliable diagnostic tools, such as real-time polymerase chain reaction based methods which have proven greater sensitivity particularly in the screening of malaria, is prominent. This study was conducted at the French National Malaria Reference Centre to assess sensitivity and specificity of two commercial malaria qPCR kits and two in-house developed qPCRs compared to LAMP.

**Methods:**

183 blood samples received for expertise at the FNMRC were included in this study and were subjected to four different qPCR methods: the Biosynex Ampliquick^®^ Malaria test, the BioEvolution *Plasmodium* Typage test, the in-house HRM and the in-house TaqMan qPCRs. The specificity and sensitivity of each method and their confidence intervals were determined with the LAMP-based assay Alethia® Malaria as the reference for malaria diagnosis. The accuracy of species diagnosis of the Ampliquick^®^ Malaria test and the two in-house qPCRs was also evaluated using the BioEvolution *Plasmodium* Typage test as the reference method for species identification.

**Results:**

The main results showed that when compared to LAMP, a test with excellent diagnostic performances, the two in-house developed qPCRs were the most sensitive (sensitivity at 100% for the in-house TaqMan qPCR and 98.1% for the in-house HRM qPCR), followed by the two commercial kits: the Biosynex Ampliquick^®^ Malaria test (sensitivity at 97.2%) and the BioEvolution *Plasmodium* Typage (sensitivity at 95.4%). Additionally, with the in-house qPCRs we were able to confirm a *Plasmodium falciparum* infection in microscopically negative samples that were not detected by commercial qPCR kits. This demonstrates that the *var* genes of *P. falciparum* used in these in-house qPCRs are more reliable targets than the 18S sRNA commonly used in most of the developed qPCR methods for malaria diagnosis.

**Conclusion:**

Overall, these results accentuate the role molecular methods could play in the screening of malaria. This may represent a helpful tool for other laboratories looking to implement molecular diagnosis methods in their routine analysis, which could be essential for the detection and treatment of malaria carriers and even for the eradication of this disease.

**Supplementary Information:**

The online version contains supplementary material available at 10.1186/s12936-022-04219-1.

## Background

Malaria is an infectious disease caused by a mosquito-transmitted parasite of the genus *Plasmodium*. According to the World Malaria Report of 2021, 241 million malaria cases were estimated in 2020 in 85 malaria endemic countries [1]. Even though the mortality rate of this disease has reduced globally through the years over the period 2000–2019, it is still considered as one of the biggest causes of mortality with an estimated 627,000 deaths in 2020 [1]. In fact, in 2020 malaria deaths increased by 12% compared to 2019 with an estimated 47,000 (68%) of the additional 69,000 deaths that were caused by service disruptions during the COVID-19 pandemic [1]. More importantly, it is one of the leading causes of death for children under five and is problematic for pregnant women in endemic countries [1].

Six common *Plasmodium* species are known to be responsible for the majority of human infections: *Plasmodium falciparum*, *Plasmodium vivax*, *Plasmodium malariae*, *Plasmodium knowlesi* and *Plasmodium ovale*, which is divided in two genetically distinct sympatric species *P. ovale curtisi* and *P. ovale wallikeri* [2]. However, recent advances in molecular diagnosis and genotyping have shown that other primate malaria species can also cause human infections including *Plasmodium brasilianum* [3], *Plasmodium simium* [4] and *Plasmodium cynomolgi* [5]. In metropolitan France, most reported cases are imported from sub-Saharan Africa. Based on the reports of the French National Malaria Reference Centre (FNMRC), 2895 malaria cases were declared in 2019, but over 5000 imported cases have been estimated in France. Symptomatic patients were mainly migrants, travellers or military staff [6].

In practice, accurate diagnosis of this disease is a very important tool for an effective treatment. The microscopic examination of Giemsa-stained thick blood smears has always been the “gold standard” for malaria diagnosis in many endemic areas [7]. This method is inexpensive and ensures the identification of *Plasmodium* species and parasite densities. However, it is limited due to the inter-observer variability especially with low parasitaemia and mixed or non-falciparum infections [8]. Therefore, it requires well-trained experts and microbiologists. In addition to light microscopy, the World Health Organization (WHO) recommends the use of immuno-chromatographic rapid diagnostic tests (RDTs) as a routine tool for malaria diagnosis [9]. These tests detect parasite antigens such as the histidine-rich protein II (HRP2) synthesized by *P. falciparum* and the *Plasmodium* specific lactate dehydrogenase (pLDH) or p-aldolase usually synthesized during the erythrocytic cycle and therefore common to all malaria species [10]. Recently, some of these RDT’s have included the *P. vivax*-specific lactate dehydrogenase (pvLDH) allowing the detection of *P. vivax* [11]. While these tests can detect approximately 100 parasites/µL (0.002% parasitaemia) [12], their interpretation can be tricky. In fact, the major restrictions of RDTs are: cases when the test is falsely interpreted as positive due to the persistence of HRP2 in the blood even several days after parasite clearance and malaria recovery [13], false negatives caused by gene deletions and decreased sensitivity for non-falciparum infections [14, 15].

Since the late 1980s, several polymerase chain reaction (PCR) based methods were developed for malaria detection. These techniques represented a significant improvement to light microscopy and other conventional diagnostic tools because of the superior limit of detection (LOD) [8]. Most of the methods developed had a common target: the *Plasmodium* 18S SSU RNA gene, including the loop-mediated isothermal amplification (LAMP), nested, semi-nested and real-time PCRs [16]. Nevertheless, the specific identification of the different *Plasmodium* species has remained problematic since it requires multiplexing, which can cause primer competition and thus failure to detect species with lower parasite densities in mixed infections. It could also be either time consuming or expensive.

The LAMP methodology was first published in the year 2000 and is based on the isothermal amplification of DNA by using the high strand displacement activity of the *Bacillus stearothermophilus (Bst)* DNA polymerase and specific sets of inner and outer primers identifying distinct regions of the targeted DNA [17]. This generates loop formations and inverted repeats of target sequences permitting a highly efficient DNA amplification under isothermal conditions in less than one hour with a LOD as few as six copies [17].

When quantitative real-time PCR (qPCR) technology was first introduced, it was considered revolutionary for the molecular diagnosis of malaria. It is in fact more sensitive than other conventional PCR methods (LOD < 0.1 parasites/µL) [18] and easier to execute with no post-PCR manipulations. In practice, two main types of qPCRs exist: the ones using fluorescent dyes such as SYBR green which intercalates with nonspecific double-stranded DNA and the ones with specific fluorescent probes such as TaqMan probes [19]. A new method has been recently added to the molecular detection and genotyping of parasites, real-time qPCR coupled with high resolution melting (HRM) curve analysis. This technique is based on detecting the differences of nucleotide sequences in targeted fragments of a gene generating different melting temperatures (Tm) by amplifying the region of interest in the presence of a specialized DNA binding dye and gradual denaturation of the amplicons producing characteristic melting profiles [19]. This method has already been used for both *Plasmodium* diagnosis and distinction and was proven to be very effective by Chua et al. and Joste et al. [19, 20].

The present study was carried out at the French National Malaria Reference Centre (FNMRC) with the aim to assess the sensitivity and specificity of a new commercial malaria qPCR kit, the Ampliquick® Malaria test (Biosynex), and two in-house qPCRs developed at the FNMRC with the LAMP Alethia^®^ Malaria (Meridian Bioscience) as the reference method for positivity. The LAMP technology has shown ≥ 95% pooled sensitivity and specificity for the detection of *Plasmodium* infections and is, therefore, deemed to be a test with an excellent diagnostic performance [21]. This test is also the first screening option used in some medical laboratories in France. For those reasons, the commercial LAMP-based assay Alethia^®^ Malaria was considered as the gold standard for sensitivity during this study. However, for the identification of malaria species, the three tested qPCR methods were compared with the TaqMan qPCR *Plasmodium* Typage (Bio-Evolution), another commercial kit available in France.

## Methods

### Clinical samples

For this study, blood samples of patients suspected with malaria and received for expertise at the FNMRC from January 2019 to January 2020 were retrospectively included. These blood samples were collected into EDTA tubes and, after reception, were subjected to routine biological diagnosis: microscopy on stained thin and thick blood films and DNA extraction.

There was no need for specific consent from the patients since all the data was collected from the FNMRC’s database and analysed in accordance with the common public health mission of all the National Reference Centres of France. Everything was coordinated with the ‘Santé Publique France’ organization for malaria surveillance and care. According to the article L1221-1.1 of the public health code in France, the study of biological samples obtained from routine medical care is considered as non-interventional research and therefore only requires the non-opposition of the patient during sampling (per article L1211-2 of the public health code). The data collected were anonymized before use.

### LAMP method

The commercial LAMP based assay Alethia^®^ Malaria is a qualitative isothermal molecular test allowing the direct detection of *Plasmodium* spp, without species identification, by targeting segments of its mitochondrial DNA after lysis of whole blood samples. It was performed on the Illumipro-10™ (Meridian Bioscience, Ohio, US) automated isothermal amplification and detection system following the test procedure provided by the manufacturer.

### DNA extraction

DNA was extracted from a sample of 200 µL of whole blood and eluted in 100 µL of buffer using Magnapure^®^ (Roche diagnosis, Bale, Switzerland) following the manufacturer’s instructions. DNA was then stored at − 20 °C until analyses.

### *Plasmodium* Typage qPCR method

The *Plasmodium* Typage (Bio-Evolution, Île-de-France, France) real-time qPCR kit is a TaqMan based diagnostic test allowing the detection and the simultaneous identification of *P. falciparum*, *P. ovale*, *P. vivax*, *P. malariae* and *P. knowlesi*. This test was routinely used for malaria diagnosis at the FNMRC.

Two reaction wells are necessary to detect the five *Plasmodium* species each one containing 15 µL of either the Master Mix 1 or the Master Mix 2, prepared following the procedure provided by the manufacturer, and 5 µL of extracted DNA. Ready to use positive and negative controls are also provided with the qPCR kit. Primers targeting the human *beta actin* are used in the Master Mix 1 to control DNA extraction. This qPCR was performed using the Viia7™ (Thermo Fisher Scientific, Massachusetts, US) thermal cycler following the thermal program: 30 s at 95 °C then 40 cycles of 15 s at 95 °C and 45 s at 60 °C and finally a cooling phase of 1 s at 37 °C.

### Ampliquick^®^ Malaria qPCR method

The Ampliquick^®^ Malaria kit is a real-time TaqMan qPCR diagnostic test. This technique depends on the gene amplification of a specific region of the 18S RNA gene of *Plasmodium* spp (Pan) and *P. falciparum*. This kit can be used in two ways: directly from a whole blood sample following the manufacturer’s instructions or after DNA extraction. The whole blood sample direct detection protocol was not tested in this study.

qPCR experiments were performed using the 7500 Fast Real-Time PCR System (Thermo Fisher Scientific, Massachusetts, US) following the thermal program: 3 min at 95 °C then 50 cycles of 10 s at 94 °C and 20 s at 60 °C. Each reaction well contained 8 µL of sample or control and 12 µL of the lyophilized mix in microtubes provided by the manufacturer.

### In-house HRM and TaqMan qPCR assays development

The primers used for both the HRM and TaqMan assays, described by Schindler et al. [22], target two independent *Plasmodium* genes: the *Pan-Plasmodium* 18S rRNA sequence (Pspp 18S) and the *P. falciparum-*specific acidic terminal sequence of the *var* genes (PfvarATS). The HsRNaseP human gene was used as an internal control (Ci) to rule out extraction failure (Table 1).

**Table 1:** The mix of primers and probes used in this study, adapted from Schindler et al. [22]

Species	Oligo name	Target region	Oligo final concentrations (TaqMan assay) (µM)	Oligo final concentrations (HRM assay) (µM)	Supplier
*Pan-Plasmodium*	Pspp18S fwd	18S rRNA	0.4	0.3	Eurogentec
	Pspp18S rev	18S rRNA	0.4	0.3	Eurogentec
	Pspp18S probe	18S rRNA	0.2	–	Eurogentec
*P. falciparum*	PfvarATS fwd	varATS	0.4	0.3	Eurogentec
	PfvarATS rev	varATS	0.4	0.3	Eurogentec
	PfvarATS probe	varATS	0.25	–	Eurogentec
*H. sapiens* (Ci)	HsRNaseP fwd	Rnasep gene	0.2	–	Eurogentec
	HsRNaseP rev	Rnasep gene	0.2	–	Eurogentec
	HsRNaseP probe	Rnasep gene	0.1	–	Eurogentec

The HRM and TaqMan qPCR assays were performed on the Viia7™ Real-Time PCR System (Thermo Fisher Scientific, Massachusetts, US). The thermal profile for the HRM-qPCR was as following: initialization step at 95 °C for 10 min, 40 cycles of 15 s at 95 °C, 1 min à 60 °C and an HRM phase of 10 s at 95 °C and 1 min at 60 °C then 15 s at 95 °C and 15 s at 60 °C. The one for the TaqMan-qPCR was: 15 min at 95 °C, 45 cycles of 15 s at 95 °C and 1 min at 55 °C.

The reaction wells for the HRM assay contained 10 µL of the 1 × MeltDoctor™ HRM Master Mix (Thermo Fisher Scientific, Massachusetts, US), 0.3 µM of each primer (Pspp18S and PfvarATS) (Table 1) and 5 µL DNA. The reaction wells for the TaqMan assay contained 10 µL of the 1 × Luna^®^ Universal Probe qPCR Master Mix (New England BioLabs Inc, Massachusetts, US), 0.1 to 0.4 µM of the probes and primers of each target (Pspp18S, PfvarATS and HsRNaseP) (Table 1) and 6 µL DNA.

### Statistical analysis

The sensitivity, specificity and Kappa coefficient with 95% confidence intervals (CI) of each qPCR method were calculated compared to the LAMP method. The R software was used to perform statistical tests and calculate the Kappa coefficient [23]. Ct values were compared using the Mann–Whitney U-test and the linear regression was evaluated using the F-test.

## Results

### Screening of clinical samples

A total of 183 samples were included but only 147 were analysed with the LAMP Alethia^®^ Malaria method, the gold standard for positivity in this study, to determine whether there was a *Plasmodium* infection or not (Additional file 1). The results showed 104 LAMP positive samples from which only 86 were positive by microscopy, and 43 negatives.

For the remaining 36 *Plasmodium* spp. positive samples included in this study, but not tested with the LAMP method, DNA extracts were still used to compare species diagnosis and to correlate the cycle thresholds (Ct) and the parasite density for the different qPCR methods.

### Average melting curve peak (Tm) values for *Plasmodium* spp. *and P. falciparum*

**Fig. 1:**
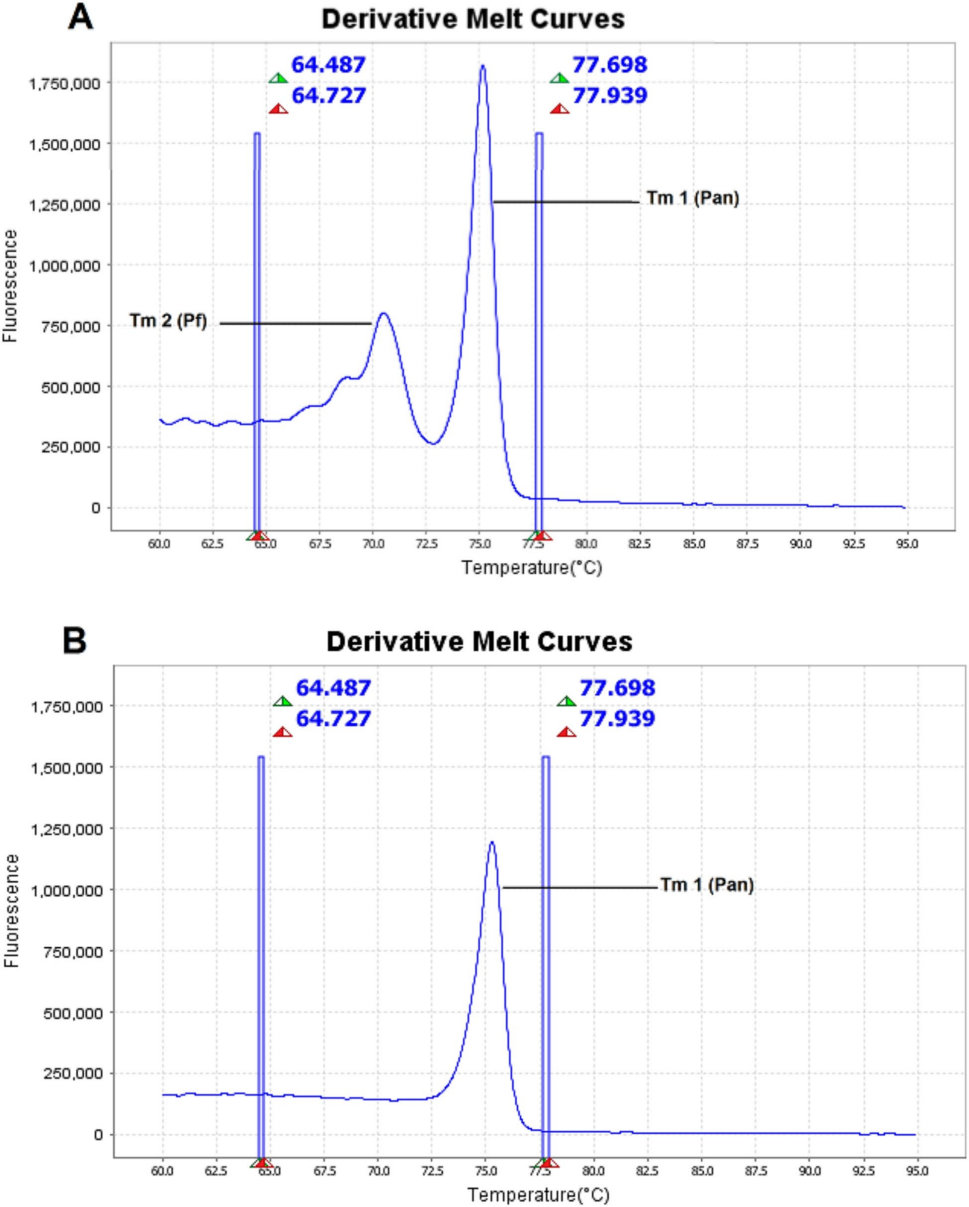
Derivative Melt curve peaks (Tm) for *P. falciparum* and *Plasmodium* spp. after HRM phase. **A** Tm peaks for both *P. falciparum* and *Plasmodium* spp. targets in case of *P. falciparum* carriage. **B** Tm peak for the *Plasmodium* spp. target in case of *Plasmodium* spp. other than *P. falciparum* carriage. X axis represents the temperature (°C). Y axis represents the fluorescence (derivative melt curve)

The in-house HRM-qPCR assay was able to identify the presence of a *Plasmodium* spp. or a *P. falciparum* infection. *Plasmodium* spp infections displayed a specific Pan melting curve peak value (Tm1), which was reproducible no matter the species or the association of species. In case of a *P. falciparum* infection, a second Tm value specific to *P. falciparum* (Tm2) was also present (Table 2). As shown in Fig. 1, the Tm of *P. falciparum* can be clearly distinguished from that of *Plasmodium* spp. (Pan).

**Table 2:** Average Tm values and their Standard Deviations (SD) for *P. falciparum* and *Plasmodium* spp other than *P. falciparum*

	*P. falciparum* infection	Non- *P. falciparum* infection
Tm 1 (Pan target) ± SD	75,258 ± 0,458	75,260 ± 0,458
Tm 2 (Pf target) ± SD	70,825 ± 0,250	–

### Evaluation of the sensitivity and specificity of the qPCR methods

The 147 isolates tested with LAMP were assessed with the four different qPCR methods. The 43 LAMP negative samples were also negative by all four methods, which gives them all 100% specificity. Of the 104 positive samples, five were not detected by *Plasmodium* Typage (Bio-Evolution), three by Ampliquick^®^ Malaria and two by the HRM-qPCR but all with the in-house TaqMan-qPCR (Table 3).

**Table 3:** Sensitivity and Specificity of microscopy and the different qPCR methods studied compared to LAMP Alethia® Malaria

	Microscopy		*Plasmodium* Typage (Bio-Evolution)			Ampliquick^®^ Malaria			In-house HRM			In-house TaqMan	
	** + **	**−**	** + **	**−**	** + **		**−**	** + **		**−**	** + **		**−**
LAMP + (N = 104)	86	18	99	5	101		3	102		2	104		0
LAMP − (N = 43)	0	43	0	43	0		43	0		43	0		43
Specificity	100%		100%		100%			100%			100%		
Sensitivity 95% CI	85.2% [74–89.4]		95.4% [89.1–98.4]		97.2% [91.8–99.4]			98.1% [93–99.8]			100%[100–100]		
Kappa 95% CI	0.74 [0.63–0.85]		0.92 [0.85–0.99]		0.95 [0.9–1]			0.97 [0.92–1]			1 [1–1]		

Compared to LAMP, the in-house TaqMan and HRM qPCRs were the most sensitive (sensitivity = 100%, 95% CI [100–100] and 98.1%, 95% CI [93–99.8] respectively), followed by the two commercial kits: the Ampliquick^®^ Malaria test (sensitivity = 97.2%, 95% CI [91.8–99.4]) and the Bio-Evolution *Plasmodium* Typage (sensitivity = 95.4%, 95% CI [89.1–98.4]). Microscopic techniques had a sensitivity of 85.2% (95% CI [74–89.4]). Kappa’s coefficient shows that the qPCRs tested are highly concordant with the LAMP method, in particular the in-house qPCRs (Table 3). All positive samples by microscopy (n = 86) were also positive after analysis with the four tested qPCR methods.

### Accuracy of species diagnosis

On each included sample, microscopy diagnosis and species identification by qPCR were systematically done (Additional file 2). The Bio-Evolution *Plasmodium* Typage is a routinely used qPCR kit for malaria diagnosis at the FNMRC. It allows the simultaneous identification of *P. falciparum*, *P. ovale*, *P. vivax*, *P. malariae* and *P. knowlesi* and was therefore considered as the reference method for the *Plasmodium* species identification when the sample was positive by this method.

Analysis with the Bio-Evolution kit showed 135 positive and 48 negative samples. Of the 135 positive samples: 73 were identified as *P. falciparum,* 36 were identified as *P. ovale* spp*,* 8 were identified as *P. vivax,* 15 were identified as *P. malariae,* 2 mixed infections by *P. falciparum* and *P. malariae* and one mixed infection *P. falciparum*, *P. malariae* and *P. ovale.*

These findings were compared with the other techniques (Table 4). The results for *P. falciparum*, *P. ovale*, *P. vivax* and the mixed infections were correlated no matter the qPCR method used. Additionally, out of the 15 *P. malariae* positive samples by the Bio-Evolution kit, one turned out to be a mixed infection highlighted by the in-house TaqMan since this assay confirmed the presence of *P. falciparum* as well (Table 4).

**Table 4:** Accuracy of *Plasmodium* species identification with the different qPCR methods studied compared to *Plasmodium* Typage (Bio-Evolution)

*Plasmodium* Typage (Bio-Evolution)	Ampliquick^®^ Malaria		In-house HRM qPCR		In-house TaqMan qPCR	
*P. falciparum* (n = 73)	Pf+/Pan+	73	Pf+/Pan+	73	Pf+/Pan+	71
					Pf+	2
*P. ovale* (n = 36)	Pan+	36	Pan+	36	Pan+	36
*P. vivax* (n = 8)	Pan+	8	Pan+	8	Pan+	8
*P. malariae* (n = 15)	Pan+	15	Pan+	15	Pan+	14
					Pf+/Pan+	1
*P. falciparum* + *P. malariae* (n = 2)	Pf+/Pan+	2	Pf+/Pan+	2	Pf+/Pan+	2
*P. falciparum* + *P. malariae* + *P. ovale* (n = 1)	Pf+/Pan+	1	Pf+/Pan+	1	Pf+/Pan+	1

The five negative isolates with the Bio-Evolution *Plasmodium* typage but positive with the LAMP Alethia^®^ Malaria turned out positive (four *P. falciparum* and one *Plasmodium* spp. other than *P. falciparum*) after the in-house TaqMan-qPCR analysis, which correlated with the initial LAMP results (Table 5).

**Table 5:** Correlation between Alethia^®^ Malaria (LAMP) and the different qPCRs for the discordant samples with Bio-Evolution

Alethia^®^ Malaria (LAMP)		*Plasmodium* Typage (Bio-Evolution)		Ampliquick^®^ Malaria		In-house HRM qPCR		In-house TaqMan qPCR	
Positive	5	Negative	5	Negative	3	Negative	2	Negative	0
		Positive	0	Pf+/Pan+	2	Pf+/Pan+	1	Pf+/Pan+	3
						Pf+	1	Pf+	1
						Pan+	1	Pan+	1

Tables 4 and 5 show that for the in-house TaqMan-qPCR, the 18S rRNA target (Pan) was positive in 96% (74/77) of *P. falciparum* mono-infections. The three remaining mono-infections were only positive for the *var* genes target (*P. falciparum*).

### Correlation between parasite density and qPCR cycle threshold (Ct) values

The median Pan-*Plasmodium* Ct of the Ampliquick^®^ Malaria (median Ct = 23.40), the species Ct of the Bio-Evolution *Plasmodium* typage (median Ct = 27) and the undifferentiated Ct of the in-house HRM-qPCR (median Ct = 22.88) and the in-house TaqMan-qPCR (median Ct = 23.58) were calculated. The data show that the in-house qPCRs and the commercial Ampliquick^®^ Malaria have lower Ct values than the Bio-Evolution *Plasmodium* Typage (pooled Ct for all species) (p < 0.001, Mann–Whitney U-test), concordant with a better sensitivity. For the in-house TaqMan-qPCR, Ct of the *var* genes and the 18S rRNA targets were not different (24.2 vs 23.5, p = 0.31, Mann–Whitney U-test).

**Fig. 2:**
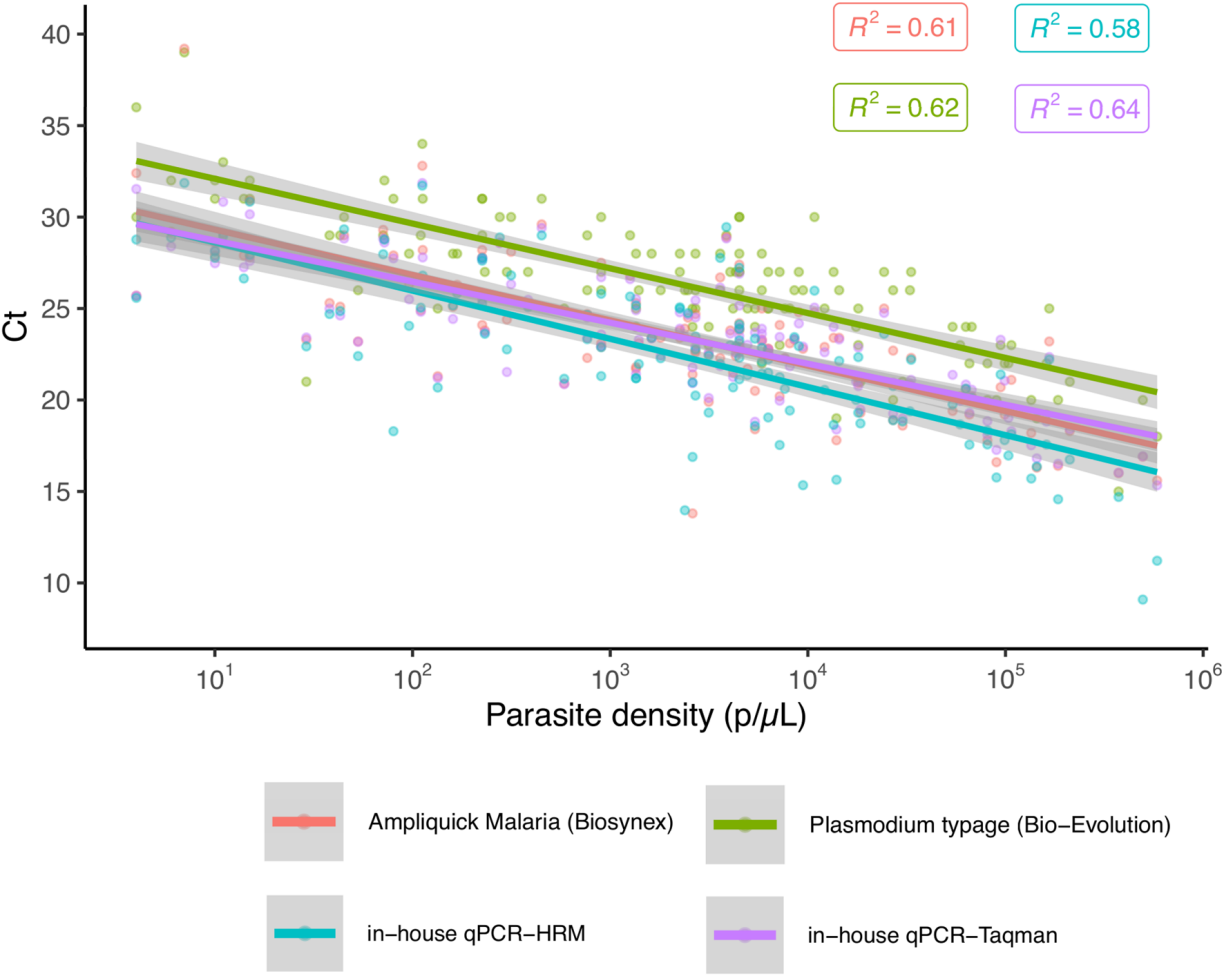
qPCR Ct values compared to parasite density. Linear regression between the parasite density and Pan-*Plasmodium* Ct of the Biosynex Ampliquick^®^ Malaria (red), the species Ct of the Bio-Evolution *Plasmodium* typage (green), the undifferentiated Ct of the in-house qPCR-HRM (blue) and the in-house qPCR-TaqMan (purple). 95% confidence interval (gray) and coefficient of determination R^2^ are indicated

As shown in Fig. 2, comparison of the parasite density determined by microscopy and the Ct values of the 135 positive samples with the different qPCR methods shows a clear correlation between the four methods and the parasite density (all linear regression p-values are < 0.001 with the F-test).

## Discussion

To control malaria, accurate and rapid diagnostic tools are very essential not only for the establishment of an effective treatment but also for surveillance and epidemiological monitoring. The quality and sensitivity of the diagnosis are very important criteria to avoid misdiagnosis which could lead to severe health problems, recrudescence, drug resistance and possibly death [24].

The implication of the molecular diagnosis of malaria has been highlighted in many studies. As reported in the literature, PCR and qPCR methods displayed high sensitivity compared to other conventional methods such as microscopy or RDTs [8, 13, 24]. In the present study, the sensitivity and specificity of the commercial malaria qPCR kit Ampliquick^®^ Malaria and two in-house developed qPCRs were assessed by comparing them with the highly sensitive LAMP method Alethia^®^ Malaria for sensibility evaluation and with the *Plasmodium* typage (Bio-Evolution) qPCR method for species diagnosis evaluation. Unlike other LAMP methods that can differentiate *Plasmodium *spp., *P. falciparum* or *P. vivax* infections [25], Alethia^®^ Malaria does not allow species diagnosis and requires, for that reason, an equally sensitive species PCR. It is very necessary to differentiate the *Plasmodium* species to identify *P. ovale* or *P. vivax* infections because they require specific treatment with primaquine, but also *P. knowlesi* infections which can lead to severe illness and are commonly misidentified with conventional methods.

The main results of this study showed that when compared to LAMP, the gold standard for positivity throughout this study, the Bio-Evolution *Plasmodium* Typage was the least sensitive at 95.2% and the in-house TaqMan-qPCR was the most sensitive at 100%. When it comes to commercial kits, it is very clear that Ampliquick^®^ Malaria is better at detecting *Plasmodium* infections than the Bio-Evolution *Plasmodium* Typage. While analysing the data, the Bio-Evolution kit results were compared to microscopy results and when the initial microscopy diagnosis is positive for a *Plasmodium* infection, the subsequent species identification by this method is accurate. Mixed infections were even distinguished in some cases. However, when compared to negative microscopy results it is clear that this qPCR method is not as reliable as the others in detecting false negatives. Indeed, out of the 61 negatives with microscopy, 48 were negative with *Plasmodium* Typage (Bio-Evolution) while 43 were considered truly negative after the LAMP and the in-house TaqMan qPCR analysis. Of the 48 negatives one was identified as a *Plasmodium* spp. infection and four were identified as *P. falciparum* mono-infections. These findings show that the in-house TaqMan qPCR was able to detect *P. falciparum* infections that were not detected by the commercial kit.

The in-house developed qPCRs target the *var* genes for *P. falciparum* detection, a multigenic family with approximately sixty copies in each *P. falciparum* genome [26]. It may probably explain the better sensitivity compared to the two commercial kits that target the 18S rRNA gene, present in four to eight copies in each *Plasmodium* genome. This was also highlighted when three samples were only positive for the *var* genes target after the in-house TaqMan qPCR. These samples were either microscopically negative or had very low parasitaemia implying that the *var* genes of *P. falciparum* are more sensitive targets than the 18S rRNA (Pan), despite no statistical difference in Ct values. Moreover, the same DNA extracts were evaluated with the four qPCR methods implying that the different sensitivities are proper to each method and not due to the quality of DNA extraction.

Compared to *Plasmodium* Typage (Bio-Evolution), the major limitation of the other techniques is the absence of species identification. The other downside is, with it being a commercial kit, the Ampliquick^®^ Malaria test is more expensive and thus cannot be systematically used in developing countries for the diagnosis of malaria. However, the TaqMan and HRM in-house qPCRs adapted from Schindler et al. [22] showed both high sensitivity and cost effectiveness.

## Conclusion

This paper represents a comparative study in which four qPCR methods for malaria diagnosis were evaluated to provide information about their sensitivities and accuracy. This could be a helpful tool for other laboratories looking to implement molecular diagnosis methods in their routine analysis. Compared to the LAMP assay, the four tested qPCR methods showed varying sensitivities with the in-house TaqMan qPCR being the most sensitive. Although it does not enable the detection of the five common malaria human infecting species, the in-house TaqMan has a comparable sensitivity to that of the LAMP assay with the advantage of identifying *P. falciparum* infections, which is the most common and life-threatening species.

Taken together, the results demonstrate the role molecular methods could play in the screening of malaria and infectious diseases in a rapid and effective way by providing critical information for the clinical context and the epidemiological survey.

## Supplementary Information

**Additional file 1. **The different methods used to evaluate the included samples. A flow-chart showing the number of samples included and the different methods we used to analyze these samples.

**Additional file 2. **Results of the microscopic diagnosis and molecular assays for each included sample. A table detailing the results of: microscopic diagnosis (species identification and parasite densities), LAMP Alethia^®^ Malaria and the four different qPCR methods tested in this study for the 183 included samples.

## Data Availability

All data generated or analysed during this study are included in this published article and its additional files.
